# Sealing ability of bioceramic versus conventional endodontic sealers: a systematic review of *in vitro* studies

**DOI:** 10.3389/fdmed.2026.1974966

**Published:** 2026-10-20

**Authors:** Venkata Suresh Venkataiah, Filippo Cardinali, William Kano, Deepak Mehta, Irina Makeeva, Stella Constantinidou, Simone Grandini, Salvatore Sauro

**Affiliations:** 1 Clinical Sciences Department, Centre of Medical and Bio-Allied Health Science Research, College of Dentistry, Ajman University, Ajman, United Arab Emirates; 2 Dental Biomaterials and Minimally Invasive Dentistry, Departamento de Odontologia, Facultad de Ciencia de la Salud, Universidad CEU-Cardenal Herrera, Valencia, Spain; 3 Department of Medical Biotechnologies, Unit of Endodontics and Restorative Dentistry, University of Siena, Siena, Italy; 4 Department of Operative Dentistry, Tohoku Graduate School of Dentistry, Tohoku University, Sendai, Japan; 5 Department of Therapeutic Dentistry, Sechenov University of Moscow, Moscow, Russia

**Keywords:** apical microleakage, bioceramic sealers, conventional sealers, endodontic obturation, root canal therapy, sealing ability

## Abstract

**Background:**

Bioceramic sealers are contemporary bioactive endodontic biomaterials that have gained considerable attention because of their favorable physicochemical and biological properties, including bioactivity and biocompatibility. These characteristics may extend their function beyond passive sealing; however, studies comparing their sealing performance with conventional sealers have reported inconsistent findings.

**Objective:**

To systematically review the available *in vitro* evidence comparing the sealing ability of bioceramic and conventional endodontic sealers.

**Methods:**

A systematic literature search was performed in PubMed, Scopus, Web of Science, and Google Scholar to identify full-text *in vitro* experimental studies published in English between January 2000 and April 2026. The search strategy used the terms (sealing effectiveness OR sealing ability) AND (bioceramic sealers OR conventional sealers). Study selection was conducted in accordance with the PRISMA 2020 guidelines. Risk of bias was assessed using a structured assessment based on domains adapted from the Cochrane RoB 2 framework for laboratory studies.

**Results:**

Twelve *in vitro* controlled laboratory studies involving 831 specimens met the inclusion criteria. Seven studies (58%) reported findings favoring bioceramic sealers, four studies (33%) favored conventional sealers, and one study (8%) found no statistically significant difference between groups. No consistent association was identified between obturation technique and sealing outcomes.

**Conclusion:**

Most included studies reported superior sealing ability with bioceramic sealers compared with conventional sealers. However, the findings were not uniform across all studies. Further well-designed *in vivo* investigations are required to determine whether these *in vitro* observations translate into clinical benefit.

**Systematic Review Registration:**

https://osf.io/v9gnc, identifier: v9gnc.

## Introduction

Effective root canal (RC) therapy is essential for preventing the colonization and proliferation of pathogenic microorganisms within the root canal system (1). A key stage of treatment is obturation, defined as the three-dimensional filling of the prepared root canal system. Inadequate obturation may permit secondary infection and compromise treatment outcomes (2). Endodontic sealers contribute to this process by filling voids between the core obturation material and canal walls, helping to establish a fluid-tight seal and limit microbial leakage (3).

Conventional endodontic sealers, particularly resin-based materials such as AH Plus and zinc oxide eugenol (ZOE) sealers, have been widely used because of their favorable physicochemical characteristics and antibacterial properties, which may support periapical healing and reduce the risk of reinfection (4). However, these materials are associated with several recognized limitations, including polymerization shrinkage or dimensional change during setting, solubility and resorption, and the potential for cytotoxic or inflammatory effects in periapical tissues, especially before complete setting has occurred (5).

Bioceramic sealers, including EndoSequence BC Sealer, TotalFill BC, and BioRoot RCS, have been introduced as an alternative class of obturation materials (6). These materials are inorganic and non-metallic and are generally characterized by chemical stability, resistance to corrosion, hydrophilicity, and physical properties intended to be compatible with dentinal tissues (7). Their ability to undergo slight expansion during setting, penetrate dentinal tubules, and form a mineralized interface along the canal wall has been suggested as a potential explanation for improved sealing performance relative to conventional sealers (7, 8). In addition, bioceramic sealers are commonly used with hydraulic single-cone obturation techniques, which may facilitate more uniform sealer distribution and simplify retreatment in selected clinical situations (9).

Many contemporary bioceramic sealers are calcium silicate-based materials whose setting reaction depends on moisture and results in the formation of calcium silicate hydrate (C-S-H) and calcium hydroxide as the principal hydration products (4, 6, 8). In the presence of tissue fluids and phosphate ions, calcium hydroxide may promote hydroxyapatite deposition at the sealer-dentin interface. This process provides a basis for chemical bonding that differs from the predominantly micromechanical adhesion associated with epoxy resin-based sealers (4, 6, 8). Because interfacial mineralization develops over time, the quality of the seal may improve progressively after obturation rather than being established immediately. Calcium silicate-based sealers have also been associated with favorable biocompatibility profiles, often attributed to calcium ion release, and this has been proposed as a potential advantage in supporting periapical tissue healing and repair (4, 6, 8).

Collectively, these properties position bioceramic sealers as contemporary bioactive endodontic biomaterials rather than solely as passive sealing materials. Their calcium ion release, biomineralization potential, biocompatibility, and interactions with dentine and periapical tissues may contribute to a local environment favorable to tissue repair and healing. These biological characteristics have also generated interest in their potential relevance to regenerative endodontics. From a minimally invasive perspective, effective sealing together with favorable biological interactions may also contribute to reducing reinfection and the need for retreatment, thereby supporting long-term preservation of the treated tooth.

The sealing performance of endodontic sealers may also be influenced by the obturation technique with which they are used. Hydraulic single-cone obturation is commonly associated with bioceramic sealers and relies substantially on sealer flow, adaptation, and interaction with dentinal walls to occupy the space between the gutta-percha cone and the prepared canal. In contrast, warm obturation techniques use thermoplasticized gutta-percha and compaction to improve adaptation to canal irregularities, although heating and compaction may also influence the physicochemical behavior and interfacial performance of the accompanying sealer.

Consequently, sealing ability may reflect an interaction between sealer formulation and obturation technique rather than the properties of the sealer alone.

Despite these proposed advantages, the available literature remains inconsistent. Several *in vitro* controlled laboratory studies have reported lower apical microleakage and improved sealing with specific bioceramic sealers (6, 7, 10, 13–15, 17). Other investigations have reported comparable performance between bioceramic and conventional sealers (1), while a smaller number have found superior sealing with conventional materials (3, 11). Given these conflicting findings, a systematic synthesis of the available evidence is warranted to evaluate the comparative sealing ability of bioceramic and conventional sealers and to support evidence-based material selection in endodontic practice.

The primary objective of this systematic review was to compare the sealing ability of bioceramic endodontic sealers with that of conventional endodontic sealers in *in vitro* studies. Secondary objectives were: (i) to assess whether the choice of obturation technique modifies the comparative sealing effectiveness of the two sealer categories; and (ii) to identify clinical challenges associated with the use of each sealer type.

## Materials and methods

### Review protocol and PICOS question

This systematic review was conducted and reported in accordance with the Preferred Reporting Items for Systematic Reviews and Meta-Analyses (PRISMA) 2020 guidelines (18). This systematic review protocol was retrospectively registered with the Open Science Framework (OSF). Registration: https://osf.io/v9gnc**.** The review addressed the following PICOS question: In extracted teeth used for *in vitro* root canal simulation (Population), do bioceramic endodontic sealers (Intervention), compared with conventional endodontic sealers (Comparator), provide superior sealing ability (Outcome) in controlled *in vitro* experimental studies (Study design)? The PICOS framework used to define study eligibility is presented in Table 1.

**Table 1:** PICOS framework for study eligibility.

PICOS component	Definition
Population (P)	Extracted teeth used for *in vitro* root canal simulation.
Intervention (I)	Bioceramic endodontic sealers.
Comparator(C)	Conventional endodontic sealers.
Outcome (O)	Sealing ability assessed using measures of apical or interfacial leakage and sealing performance, including dye penetration, fluid filtration, micro-computed tomography (micro-CT), and related measures of sealer adaptation or void formation.
Study design (S)	Controlled *in vitro* experimental studies directly comparing bioceramic and conventional endodontic sealers.

### Search strategy

A systematic literature search was performed in PubMed, Scopus, Web of Science, and Google Scholar. The following Boolean search strategy was used: (sealing effectiveness OR sealing ability) AND (bioceramic sealers OR conventional sealers). The search was limited to studies published in English between January 2000 and April 2026. No restrictions were placed on the country of publication. The reported search strategy represents the complete combination of search terms used for the review. The relatively focused search yield reflects the specific combination of sealing-related terms and direct comparison between bioceramic and conventional sealers, together with the predefined publication period and English-language restriction. To supplement the electronic search and reduce the possibility of missing relevant studies, the reference lists of eligible studies were also screened manually.

### Inclusion and exclusion criteria

Studies were included if they: (i) were *in vitro* experimental studies comparing the sealing effectiveness of bioceramic and conventional endodontic sealers; (ii) were published in English between 2000 and 2026; and (iii) were available as full-text articles. Studies were excluded if they were *in vivo* investigations, non-experimental studies including narrative reviews and cross-sectional surveys, or conference abstracts.

### Study selection

Study selection was carried out in three stages. First, records retrieved from all databases were combined and duplicate records were removed. Second, titles and abstracts were screened according to the inclusion and exclusion criteria. Third, the full texts of potentially eligible studies were reviewed for final inclusion. Title and abstract screening and full-text eligibility assessment were performed independently by two reviewers. Disagreements were resolved through discussion between the two reviewers; when consensus could not be reached, a third reviewer served as the deciding reviewer. Reference lists of the included studies were also screened to identify additional relevant studies not captured by the electronic database search. The number of studies identified, screened, and included at each stage is shown in the PRISMA flow diagram (Figure 1).

**Figure 1:**
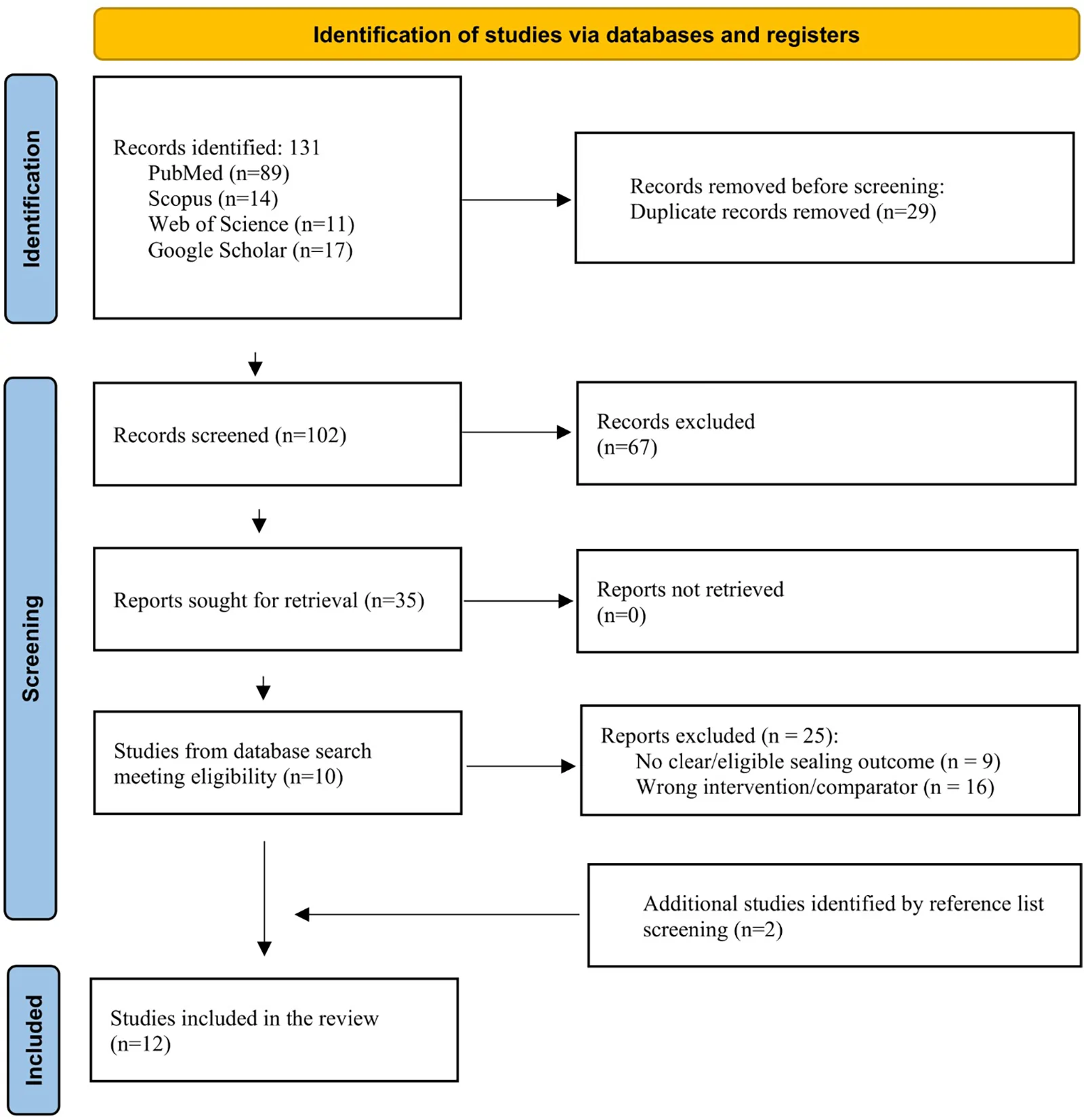
PRISMA 2020 flow diagram of study selection. Flow diagram illustrating the identification, screening, eligibility assessment, and inclusion of studies in the systematic review. A total of 131 records were identified through database searching. After duplicate removal and title/abstract screening, 35 articles underwent full-text assessment. Ten studies met the eligibility criteria, and two additional studies were identified through manual reference screening, resulting in 12 studies included in the final review.

### Data extraction

The following information was extracted from each included study: authors and year of publication, country of origin, study aim, tooth type and specimen count, sealer brands compared, obturation technique(s), outcome measures used (including dye penetration, fluid filtration, and micro-computed tomography [micro-CT]), and the main findings related to sealing ability. Data extraction was performed independently by two reviewers. Any discrepancies were resolved through discussion, with a third reviewer serving as the deciding reviewer when consensus could not be reached.

### Data synthesis

Owing to substantial methodological and outcome heterogeneity among the included studies, quantitative meta-analysis was not considered appropriate, and the findings were synthesized qualitatively. Studies differed in tooth and specimen characteristics, canal preparation and irrigation protocols, sealer formulations and comparators, and obturation techniques. In addition, sealing outcomes were assessed using different methods, including dye penetration, fluid filtration, confocal microscopy, and micro-CT. These approaches evaluate different aspects of leakage, void formation, and interfacial adaptation and did not provide sufficiently comparable effect measures for meaningful quantitative pooling.

### Methodological appraisal and risk of bias assessment

The methodological quality of the included studies was evaluated using a structured appraisal based on selected domains adapted from the Cochrane Risk of Bias 2 (RoB 2) framework. The original RoB 2 tool was not applied directly because it was developed for randomized clinical trials and has not been validated for *in vitro* laboratory studies. Instead, selected domains considered relevant to controlled laboratory experiments were adapted to provide a structured assessment of potential sources of bias.

Five domains were assessed: (D1) specimen allocation; (D2) deviations from the intended experimental procedures; (D3) outcome measurement; (D4) missing outcome data; and (D5) selective reporting of results. For each domain, a low-risk judgment was assigned when the relevant methodological feature was adequately described and no important source of bias was identified; some concerns were assigned when reporting was incomplete or a potential source of bias could not be excluded; and high risk was assigned when the reported methodology indicated a substantial potential for bias within that domain.

An overall judgment was subsequently assigned after consideration of the pattern and severity of concerns across the five domains rather than by calculation of a numerical score. Because this adapted approach has not been validated specifically for *in vitro* studies, the resulting judgments should be interpreted as a structured methodological appraisal rather than as application of the validated Cochrane RoB 2 instrument. The results of the methodological appraisal are presented in Table 4.

## Results

### Search outcomes and study selection

The database search identified 131 records. After duplicate removal, 102 unique records remained. Screening of titles and abstracts excluded 67 records that did not meet the review criteria. Full-text assessment of the remaining 35 articles resulted in the inclusion of 10 studies. Screening of the reference lists of these studies identified two additional eligible articles, resulting in a final sample of 12 studies (Figure 1). Reasons for exclusion during full-text review included inappropriate interventions, inappropriate comparators, or outcomes unrelated to sealing ability.

### Characteristics of included studies

All 12 included studies were controlled *in vitro* laboratory experiments, comprising a total of 831 specimens. Most studies used extracted mandibular and maxillary molars, premolars, and canines. Miri et al. used only single-rooted maxillary teeth (12), whereas El Sayed and Al Husseini included both maxillary and mandibular canines (13). Outcome assessment methods included fluid filtration, dye penetration using methylene blue or India ink, confocal microscopy, and micro-CT (6, 7, 10–17).

The single-cone technique was the most frequently used obturation method. Three studies also evaluated continuous wave compaction and cold lateral condensation, allowing comparisons between obturation techniques (3, 15, 16). A wide range of bioceramic and conventional sealers were represented. The most commonly investigated bioceramic sealers were EndoSequence BC Sealer, TotalFill BC, and the mineral trioxide aggregate (MTA)-based sealer MTA Fillapex (1, 7, 10, 11, 13, 16, 17). Additional bioceramic sealers included Endoseal MTA, Hybrid

Root SEAL, Fill Root ST, AH Plus Bioceramic Sealer, GuttaFlow Bioseal, BioRoot RCS, DiaRoot BC, CeraSeal, BioCera BC, SmartPaste Bio (3, 6, 10–17). AH Plus was the most frequently used conventional comparator (1, 7, 10, 11, 13, 14, 17). Other conventional sealers included Sealapex, ADseal, Tubli-Seal, AH 26, Epiphany, and GuttaFlow 2 (3, 6, 7, 12, 14, 16). The characteristics and principal findings of the included studies are summarized in Table 2, while the comparative findings for the principal sealers evaluated are summarized in Table 3.

**Table 2:** Characteristics and principal findings of the included *in vitro* studies comparing the sealing ability of bioceramic and conventional endodontic sealers.

Study	Sample	Bioceramic Sealer(s)	Conventional Comparator(s)	Main Finding
Akhtar et al. (2023) (3)	110 single-rooted mandibular premolars	EndoSequence, Endoseal	Sealapex	Significant differences by sealer type but not obturation technique. Sealapex showed the lowest apical microleakage overall. EndoSequence performed best with continuous-wave compaction and Endoseal with the single-cone technique.
Hasnain et al. (2017) (10)	74 mandibular premolars	TotalFill BC, Hybrid Root SEAL	AH Plus	TotalFill BC demonstrated the best apical sealing ability. Hybrid Root SEAL was the least effective.
Ismail et al. (2026) (6)	30 artificial root canals	Fill Root ST, AH Plus Bioceramic	ADseal	AH Plus Bioceramic produced the fewest voids and lowest film thickness. ADseal showed the greatest void formation.
Kaul et al. (2021) (1)	125 mandibular molars	EndoSequence, GuttaFlow Bioseal	AH Plus	No statistically significant differences among groups. None achieved a completely fluid-tight apical seal.
Pontoriero et al. (2021) (15)	80 single-rooted premolars	BioRoot RCS	ZOE pulp canal sealer	BioRoot RCS demonstrated superior sealing ability under both warm and cold obturation techniques.
Nimiya et al. (2025) (17)	17 single-rooted mandibular premolars	DiaRoot BC, CeraSeal, BioCera BC	AH Plus	CeraSeal showed the highest sealing ability. AH Plus outperformed DiaRoot BC and BioCera BC.
Chelja et al. (2025) (16)	25 mandibular premolars	CeraSeal, MTA Fillapex	GuttaFlow 2	Leakage occurred in all groups. GuttaFlow 2 with single-cone obturation showed the best sealing performance.
Mittal et al. (2025) (14)	62 single-rooted premolars	SmartPaste Bio	Tubli-Seal, AH Plus, RealSeal SE	SmartPaste Bio demonstrated superior sealing performance and dentinal wall adaptation.
Rizvi et al. (2021) (11)	32 teeth (incisors, canines, premolars)	MTA Fillapex	AH Plus	AH Plus exhibited significantly better apical sealing than MTA Fillapex.
Pawar et al. (2014) (7)	75 single-rooted permanent teeth	EndoSequence	AH Plus, Epiphany	EndoSequence and Epiphany demonstrated better sealing ability than AH Plus.
Miri et al. (2020) (12)	104 single-rooted maxillary teeth	TotalFill BC	AH 26	TotalFill BC exhibited significantly greater microleakage than AH 26. Calcium hydroxide dressing further increased leakage.
El Sayed & Al Husseini (2018) (13)	80 maxillary and mandibular canines	EndoSequence BC, MTA Fillapex	AH Plus	EndoSequence BC demonstrated the lowest apical microleakage among all tested sealers.

**Table 3:** Summary of the principal endodontic sealers evaluated in the included studies.

Sealer/material	Sealer category	Conventional comparator(s)	Summary of findings in the included studies
EndoSequence BC Sealer	Bioceramic	Sealapex, AH Plus, Epiphany	Performance varied among studies. EndoSequence performed best with continuous-wave compaction in the study by Akhtar et al., although Sealapex showed the lowest overall apical microleakage. Kaul et al. found no significant difference between EndoSequence, GuttaFlow Bioseal, and AH Plus. Pawar et al. reported better sealing with EndoSequence and Epiphany than with AH Plus.
TotalFill BC	Bioceramic	AH Plus, AH 26	Hasnain et al. reported superior apical sealing with TotalFill BC compared with the other sealers evaluated. In contrast, Miri et al. reported greater microleakage with TotalFill BC than with AH 26, and a calcium hydroxide dressing further increased leakage.
BioRoot RCS	Bioceramic	Zinc oxide–eugenol (ZOE) pulp canal sealer	Demonstrated superior sealing compared with the conventional ZOE sealer under both cold and warm obturation techniques.
CeraSeal	Bioceramic	AH Plus, GuttaFlow 2	Nimiya et al. reported the highest sealing ability with CeraSeal among the evaluated sealers. Chelja et al. observed leakage across all groups, with performance also influenced by the sealer– obturation combination.
MTA Fillapex	Bioceramic/MTA-based	AH Plus, GuttaFlow 2	Findings were inconsistent. Rizvi et al. found significantly better apical sealing with AH Plus than with MTA Fillapex. El Sayed and Al Husseini found that EndoSequence BC had lower leakage than MTA Fillapex. Leakage was also observed with MTA Fillapex in the comparison by Chelja et al.
Endoseal EndoSeal/MTA	Bioceramic/MTA-based	Sealapex, AH Plus	Endoseal performed best with the single-cone technique in Akhtar et al., although Sealapex exhibited the lowest overall microleakage.
AH Plus Bioceramic	Bioceramic	ADseal	In the micro-CT evaluation by Ismail et al., AH Plus Bioceramic showed fewer voids and lower film thickness, whereas ADseal demonstrated greater void formation.
GuttaFlow Bioseal	Bioceramiccontaining/bioactive sealer	AH Plus	Kaul et al. reported no statistically significant difference among GuttaFlow Bioseal, EndoSequence, and AH Plus; none achieved a completely fluid-tight apical seal.
DiaRoot BC/BioCera BC	Bioceramic	AH Plus	Both performed less favorably than CeraSeal in the study by Nimiya et al.; AH Plus also outperformed DiaRoot BC and BioCera BC.
SmartPaste Bio	Bioceramic	Tubli-Seal, AH Plus, RealSeal SE	Demonstrated superior sealing performance and dentinal wall adaptation in the study by Mittal et al.
AH Plus	Epoxy resin-based conventional sealer	Multiple bioceramic sealers	Frequently used as the conventional comparator. Its relative performance varied: it outperformed MTA Fillapex, DiaRoot BC, and BioCera BC in individual comparisons, whereas several bioceramic sealers performed better or showed comparable sealing in other studies.
AH 26	Resin-based conventional sealer	TotalFill BC	Demonstrated lower microleakage than TotalFill BC in the study by Miri et al.
Sealapex	Conventional sealer	EndoSequence, Endoseal	Produced the lowest overall apical microleakage in the comparison by Akhtar et al.
ZOE pulp canal sealer	Zinc oxide–eugenol conventional sealer	BioRoot RCS	Showed inferior sealing compared with BioRoot RCS under both warm and cold obturation conditions.
GuttaFlow 2	Silicone-based conventional sealer	CeraSeal, MTA Fillapex	Leakage occurred across the evaluated groups; the study reported that sealing performance depended on the sealer–obturation combination.
ADseal	Resin-based conventional sealer	AH Plus Bioceramic, Fill Root ST	Demonstrated greater void formation than the other evaluated sealers in the micro-CT study.
Epiphany/RealSeal SE/Tubli-Seal	Conventional comparator sealers	EndoSequence/SmartPaste Bio	Used as conventional comparators in individual studies. Epiphany performed favorably with EndoSequence relative to AH Plus, whereas SmartPaste Bio showed better sealing and adaptation than its conventional comparators.

### Sealing ability outcomes in studies favoring bioceramic sealers

Seven studies reported superior sealing ability with bioceramic sealers (6, 7, 10, 13–15, 17). Hasnain et al. reported significantly lower apical microleakage with TotalFill BC than with Hybrid Root SEAL and AH Plus and suggested that particle size, hydrophilicity, and low contact angle contributed to this performance (10). Pontoriero et al. compared BioRoot RCS with a ZOE pulp canal sealer using both cold (single-cone) and warm (vertical condensation) obturation techniques and found superior sealing with the bioceramic sealer under both conditions (15). El Sayed and Al Husseini reported lower dye leakage with EndoSequence than with MTA Fillapex (13). Nimiya et al. reported that CeraSeal exhibited the highest sealing ability among the sealers evaluated. Although the conventional sealer AH Plus outperformed DiaRoot BC and BioCera BC, its performance remained inferior to that of CeraSeal, resulting in an overall finding favoring the bioceramic sealer (17).

### Sealing ability outcomes in studies favoring conventional sealers

Four studies reported findings favoring conventional sealers (3, 11, 12, 16). Akhtar et al. compared single-cone, continuous-wave compaction, and cold lateral condensation techniques and found that Sealapex outperformed both EndoSequence and Endoseal across all techniques (3). Rizvi et al. reported a better apical seal with AH Plus than with MTA Fillapex (11). Miri et al. reported greater microleakage with TotalFill BC than with AH 26 (12). Chelja et al. observed leakage in all groups, with GuttaFlow 2 used with single-cone obturation showing the best sealing performance (16).

### Sealing ability outcomes in studies reporting no significant difference

One study found no statistically significant difference in sealing effectiveness between bioceramic and conventional sealers (1). Kaul et al. compared EndoSequence, GuttaFlow Bioseal, and AH Plus and reported that none of the materials achieved a completely fluid-tight apical seal (1).

### Effect of obturation technique

Three studies compared sealing outcomes across multiple obturation techniques (3, 15, 16). No consistent advantage was identified for any specific technique. Akhtar et al. found no statistically significant differences in sealing ability attributable to obturation technique (3). Pontoriero et al. reported superior performance of BioRoot RCS compared with the ZOE sealer under both cold and warm obturation methods (15). Chelja et al. reported leakage in all groups regardless of technique (16).

### Risk of bias

The risk-of-bias assessment is presented in Table 4. Six studies were judged to be at low overall risk of bias, five raised some concerns, and one study was judged to be at high overall risk of bias. Randomization of specimen allocation and missing outcome data were generally adequately addressed across the included studies. Some concerns arose primarily from deviations from the intended experimental interventions, outcome measurement, and selective reporting. Miri et al. was judged to be at high overall risk of bias because of concerns relating to deviations from the intended intervention.

**Table 4:** Risk-of-bias assessment of the included studies.

Study	D1	D2	D3	D4	D5	Overall
Akhtar et al. (2023) (3)	Low	Low	Low	Low	Some concerns	Some concerns
Hasnain et al. (2017) (10)	Low	Low	Low	Low	Low	Low
Ismail et al. (2026) (6)	Low	Some concerns	Low	Some concerns	Some concerns	Some concerns
Kaul et al. (2021) (1)	Low	Low	Low	Low	Low	Low
Pontoriero et al. (2021) (15)	Low	Low	Low	Low	Some concerns	Some concerns
Nimiya et al. (2025) (17)	Low	Some concerns	Low	Low	Low	Some concerns
Chelja et al. (2025) (16)	Low	Low	Low	Low	Some concerns	Some concerns
Mittal et al. (2025) (14)	Low	Low	Low	Low	Low	Low
Rizvi et al. (2021) (11)	Low	Low	Low	Some concerns	Low	Some concerns
Pawar et al. (2014) (7)	Low	Low	Low	Low	Low	Low
Miri et al. (2020) (12)	Low	High	Low	Low	Low	High
El Sayed & Al Husseini (2018) (13)	Low	Low	Low	Low	Low	Low

**Domains:** D1, randomization of specimen allocation; D2, deviations from intended experimental interventions; D3, outcome measurement; D4, missing outcome data; D5, selective reporting of results.

**Judgments:** Low risk; Some concerns; High risk.

## Discussion

This systematic review synthesized evidence from 12 *in vitro* laboratory studies comparing the sealing ability of bioceramic and conventional endodontic sealers. Seven of the 12 studies (58%) reported findings favoring bioceramic sealers, whereas four (33%) favored conventional sealers and one (8%) found no significant difference between groups. Taken together, the findings suggest that several bioceramic sealers may provide improved sealing compared with conventional materials, although this advantage was not consistent across all products or experimental conditions (6, 7, 10, 13–15, 17). These findings are broadly consistent with previous reviews. Elmsmari et al. reported higher clinical success rates at 12 and 18 months for singlecone bioceramic obturation in the limited number of available clinical studies (9). In contrast, Rekha et al., in a review of 24 studies, found no statistically significant overall difference in sealing ability and emphasized substantial variation in materials, methodologies, and outcome measures across studies (5). The available evidence therefore supports a potential advantage for some bioceramic sealers, but does not justify considering the entire material category superior to conventional sealers.

The performance of bioceramic sealers is often attributed to their material properties. Most are hydrophilic and undergo moisture-dependent setting reactions, characteristics that may improve adaptation to dentinal walls and penetration into dentinal tubules while reducing interfacial gaps associated with shrinkage-related effects observed with some resin-based materials (10). Their capacity to interact with hydroxyapatite and form mineralized interfacial layers has also been proposed as a mechanism that may reduce microleakage and improve adhesion (8). Several of the included studies used these mechanisms to explain their findings. For example, the favorable performance of TotalFill BC relative to methacrylate- and epoxy resin-based sealers was attributed to these material characteristics (10). Nevertheless, favorable physicochemical properties alone do not ensure superior sealing, as performance is also influenced by material formulation, canal conditions, and the technique used during obturation.

An important finding of this review is that bioceramic sealers should not be considered a homogeneous category. The group includes MTA-based, calcium silicate-based, and bioactive glass-containing materials with distinct setting reactions, flow characteristics, solubility profiles, and interfacial behavior. This variability was evident among the included studies. MTA Fillapex performed less favorably than AH Plus in one investigation (11), whereas EndoSequence outperformed MTA Fillapex in another (13). In a separate comparison, CeraSeal demonstrated better performance than the two other bioceramic formulations evaluated, DiaRoot BC and BioCera BC (17). These findings indicate that the classification of a material as “bioceramic” does not in itself predict its sealing performance. Comparisons should therefore focus on individual formulations rather than assuming equivalent behavior across all bioceramic sealers.

Methodological variation among the included studies likely contributed to the inconsistent findings. Differences were present in tooth selection, canal preparation protocols, irrigation procedures, obturation techniques, and methods used to assess leakage, including dye penetration, fluid filtration, and micro-CT (1, 3, 6, 7, 10–17). These methods evaluate different aspects of the sealer–canal interface and may differ in their sensitivity to voids, interfacial defects, and leakage pathways. Consequently, results obtained using one method cannot necessarily be considered equivalent to those obtained using another. Variability in specimen anatomy and experimental procedures further limits direct comparison across studies. The absence of a standardized *in vitro* protocol for evaluating sealing ability therefore remains an important limitation of the available evidence and complicates meaningful quantitative synthesis.

Obturation technique appeared to have less influence on sealing outcomes than sealer composition. Although the single-cone technique was used most frequently, studies comparing multiple techniques did not identify a consistent pattern favoring any particular approach (3, 15, 16). For hydraulic sealers that rely on moisture, flow, and interfacial interaction, material properties may have a greater influence on sealing performance than the degree of compaction achieved during obturation. This interpretation is consistent with previous suggestions that bioceramic sealers may compensate for some limitations of minimally compacted obturation approaches when used appropriately (9). Nevertheless, the interaction between sealer formulation and obturation technique remains relevant, and the performance of a particular material should ideally be evaluated using the technique for which it is intended.

Practical considerations may also influence the translation of laboratory findings into clinical practice. A cross-sectional survey reported that 54.5% of dentists used bioceramic sealers, indicating substantial adoption while also demonstrating that many clinicians continue to rely on alternative materials (19). Cost, availability, handling characteristics, and retreatability are additional practical considerations that may influence routine use. Therefore, clinical decision-making regarding sealer selection should be guided by considerations beyond *in vitro* assessments of sealing ability.

One finding of particular clinical interest relates to calcium hydroxide intracanal medication. Miri et al. investigated the effect of calcium hydroxide dressing on subsequent sealing performance and reported increased apical microleakage with the bioceramic sealer evaluated following calcium hydroxide application (12). Residual calcium hydroxide may interfere with sealer–dentin adaptation, alter dentin surface characteristics, or affect the local conditions required for hydration and interfacial mineralization. Because calcium hydroxide remains widely used during multi-visit endodontic treatment, especially in teeth with large periapical lesions, its interaction with subsequently placed sealers warrants consideration. The findings also emphasize the importance of adequate removal of intracanal medicaments before obturation, particularly when the performance of the selected sealer depends on interaction with the dentinal surface.

Laboratory findings should, however, be considered alongside the available clinical evidence. A retrospective clinical study comparing bioceramic and resin-based sealers found no statistically significant differences in clinical or radiographic outcomes (20). Similarly, a systematic review and meta-analysis of 15 prospective studies reported comparable long-term outcomes between sealer categories while noting potential advantages of premixed bioceramic sealers for postoperative pain in some situations (21). Other prospective investigations have reported improved postoperative pain scores and enhanced periapical healing with bioceramic sealers (2, 8, 22). Collectively, these findings suggest that clinical outcomes are determined by multiple interacting factors, including lesion characteristics, root canal anatomy, microbial control, patient-related variables, operator technique, obturation quality, and material selection. Differences demonstrated under controlled laboratory conditions therefore should not be assumed to translate directly into clinically meaningful advantages.

The present findings have implications for both material selection and future research. Rather than choosing a sealer solely according to whether it is classified as bioceramic or conventional, clinicians should consider the evidence supporting the individual formulation together with its handling characteristics, intended obturation technique, and the clinical circumstances in which it will be used. The variation observed among bioceramic sealers in this review is particularly important in this regard. A favorable result obtained with one formulation cannot automatically be extrapolated to another material within the same broad category.

The strengths of this review include a comprehensive search across multiple databases, study selection conducted according to PRISMA recommendations, and the use of a structured riskof-bias assessment adapted for laboratory studies. The inclusion of studies evaluating several contemporary bioceramic and conventional formulations also allowed differences within, as well as between, sealer categories to be considered. Several limitations should nevertheless be acknowledged. The review was restricted to *in vitro* evidence, and substantial methodological heterogeneity prevented meta-analysis. Differences in specimen selection, canal preparation, irrigation and obturation protocols, and methods of assessing sealing ability further limited direct comparison between studies. The inclusion of Google Scholar as a search source should also be considered a limitation because of its broader and less standardized indexing compared with bibliographic databases, which may affect the reproducibility and precision of literature retrieval. The relatively focused search strategy may also have limited retrieval of potentially relevant studies using alternative terminology, although manual screening of the reference lists of eligible studies identified additional studies not captured by the electronic search. Publication bias cannot be excluded, and the relatively small number of studies evaluating individual formulations limits the strength of product-specific conclusions. In addition, the number of commercially available bioceramic sealers continues to increase, and newer products may not yet be supported by a substantial body of evidence. Finally, the structured methodological appraisal was based on domains adapted from RoB 2 rather than a validated risk-of-bias instrument specifically developed for *in vitro* endodontic studies; the resulting judgments should therefore be interpreted with appropriate caution.

Future investigations would benefit from greater standardization of laboratory protocols, particularly with respect to specimen selection, canal preparation, irrigation, obturation procedures, ageing conditions, and methods used to quantify leakage and interfacial adaptation. Direct comparisons between specific bioceramic and established conventional sealers would provide more clinically useful information than broad comparisons between material categories. Laboratory evidence should also be complemented by adequately powered prospective clinical studies evaluating outcomes such as postoperative symptoms, periapical healing, retreatment, and long-term treatment success. Such studies are needed to determine whether differences in sealing performance observed *in vitro* translate into clinically meaningful benefits.

## Conclusion

Most of the included *in vitro* studies favored bioceramic endodontic sealers over conventional sealers, with 7 of 12 studies (58%) reporting findings favoring bioceramic sealers. No consistent advantage was identified for any particular obturation technique. However, performance varied among individual bioceramic formulations, and several studies reported either comparable or inferior results relative to conventional sealers such as AH Plus. Current clinical evidence suggests that these differences may influence outcomes such as postoperative pain and periapical healing, although the available findings remain inconsistent. Selection of endodontic sealers should therefore be based on the properties and performance of individual materials rather than on broad material classifications alone. Clinicians should also consider case-specific factors, including the potential effects of calcium hydroxide dressing in multi-visit treatment. Further well-designed randomized controlled trials with standardized outcome measures and adequate follow-up are needed to determine whether the sealing advantages observed in laboratory studies result in clinically meaningful benefits.

## Data Availability

The original contributions presented in the study are included in the article/Supplementary Material, further inquiries can be directed to the corresponding author.
